# How audience and general music performance anxiety affect classical music students’ flow experience: A close look at its dimensions

**DOI:** 10.3389/fpsyg.2022.959190

**Published:** 2022-10-28

**Authors:** Amélie J. A. A. Guyon, Horst Hildebrandt, Angelika Güsewell, Antje Horsch, Urs M. Nater, Patrick Gomez

**Affiliations:** ^1^Department of Occupational and Environmental Health, Center for Primary Care and Public Health (Unisanté), University of Lausanne, Lausanne, Switzerland; ^2^Swiss University Center for Music Physiology, Basel University of the Arts, Basel, Switzerland; ^3^Swiss University Center for Music Physiology, Zurich University of the Arts, Zurich, Switzerland; ^4^HEMU–Haute Ecole de Musique, HES-SO University of Applied Sciences and Arts Western Switzerland, Lausanne, Switzerland; ^5^Institute of Higher Education and Research in Healthcare (IUFRS), University of Lausanne, Lausanne, Switzerland; ^6^Neonatology Service, Department Woman-Mother-Child, Lausanne University Hospital, Lausanne, Switzerland; ^7^Department of Clinical and Health Psychology, University of Vienna, Vienna, Austria; ^8^University Research Platform “Stress of life (SOLE) – Processes and Mechanisms underlying Everyday Life Stress”, University of Vienna, Vienna, Austria

**Keywords:** flow state dimensions, music performance anxiety, social evaluative threat, social anxiety, classical music students

## Abstract

Flow describes a state of intense experiential involvement in an activity that is defined in terms of nine dimensions. Despite increased interest in understanding the flow experience of musicians in recent years, knowledge of how characteristics of the musician and of the music performance context affect the flow experience at the dimension level is lacking. In this study, we aimed to investigate how musicians’ general music performance anxiety (MPA) level (i.e., the general tendency to experience anxiety during solo music performances) and the presence of an audience influence the nine flow dimensions. The participants were 121 university music students who performed solo a music piece once by themselves (private performance) and once in front of an audience (public performance). Their general MPA level was measured with an adapted version of the STAI and ranged from 27 (very low MPA) to 76 (very high MPA). The level of the nine flow dimensions was assessed with the Flow State Scale-2 after each performance. The levels of “concentration on task at hand,” “sense of control,” and “autotelic experience” decreased significantly with increasing general MPA level. The levels of “unambiguous feedback” and “loss of self-consciousness” decreased significantly with increasing general MPA level during the public performance only. The level of “sense of control” was significantly lower during the public performance than the private performance across participants. The level of “unambiguous feedback” was significantly lower during the public performance than the private performance for participants with a general MPA level higher than 47. The level of “loss of self-consciousness” was significantly lower during the public performance than the private performance for participants with a general MPA level higher than 32. In contrast, the general MPA level and the audience did not significantly affect the levels of “challenge-skill balance,” “clear goals,” and “action-awareness merging.” These results show that the effects of general MPA level and audience vary greatly across flow state dimensions. We conclude that musicians’ flow state should be analyzed at the dimension level rather than as a global score. We discuss how our findings could inform the development and implementation of interventions.

## Introduction

Performing at a professional level is a demanding activity that requires high-level cognitive and sensorimotor skills coupled with aesthetic and interpretative abilities (Zatorre et al., 2007; Altenmüller and Ioannou, 2016). An important goal for musicians is performance excellence. Musicians must be able to deal with the “pressure” of public performances, which involve evaluation by the audience and other uncontrollable and unpredictable factors (Rohleder et al., 2007; Gomez et al., 2018). According to the social self-preservation theory, the combination of these elements can make music performance situations to be perceived as social-evaluative stressors threatening musician’s social self (i.e., the individual’s worth and status within a social group; Dickerson and Kemeny, 2004). In line with this framework, when musicians perform in front of an audience, they tend on average to report more anxiety, distress, nervousness, bodily complaints, and negative cognitions than when they perform without an audience (e.g., Craske and Craig, 1984; Fredrikson and Gunnarsson, 1992; Yoshie et al., 2009; Studer et al., 2012; Larrouy-Maestri and Morsomme, 2014; Fancourt et al., 2015; Chanwimalueang et al., 2017; Aufegger and Wasley, 2018). The apprehension state related to music performance is known as music performance anxiety (MPA) (Kenny, 2010; Sokoli et al., 2022). In the clinical literature, MPA is classified as a sub-category of social anxiety disorder (American Psychiatric Association, 2013). Social anxiety disorder models highlight numerous differences between non-socially and socially anxious individuals at the level of attention, interpretation, and action (Hiemisch et al., 2002; Hofmann, 2007; Nieuwenhuys and Oudejans, 2012). Compared to non-socially anxious individuals, highly socially anxious individuals exhibit an increased attention to task-irrelevant (threat-related) information, have higher social standards (i.e., expectactions and social goals), perceive poorer task-related skills, view their own emotions and bodily reactions as less controllable, and engage more in information processing that interferes with successful goal selection. MPA is an important problem among musicians, including music students (Fernholz et al., 2019). For instance, Studer et al. (2011) found that one-third of music students considered MPA a problem for themselves. Women tend to report higher MPA level than men (Hildebrandt et al., 2012; Biasutti and Concina, 2014). Musician’s proneness to experience anxiety during music performances, which we call general MPA, varies on a continuum of severity. This individual difference has been shown to have several effects on musicians’ experience. Compared to musicians with low general MPA level, musicians with high general MPA level express significantly more state anxiety, discomfort, negative self-statements, and psychosomatic symptoms when performing in front of an audience (Craske and Craig, 1984; Studer et al., 2012; Guyon et al., 2020a).

Although MPA is a common experience for many musicians, performing in front of an audience can also be associated with pleasant experiences (Beck et al., 2000; Pilger et al., 2014). In recent years, the flow experience has attracted increasing attention in the music performance literature (Biasutti, 2017; Antonini et al., 2021; Habe et al., 2021; Tan and Sin, 2021). The term “flow” describes “an almost automatic, effortless, yet highly focused state of consciousness” (Csikszentmihalyi, 1996, p. 110) experienced “when a person’s body or mind is stretched to its limits in a voluntary effort to accomplish something difficult and worthwhile” (Csikszentmihalyi, 1990, p. 3). More recently, flow has been defined as “an intrinsically rewarding state of absorption in a task in which control feels effortless” (Norsworthy et al., 2021, p. 818). In colloquial terms, the state of flow is often referred to as being “on the ball,” “in the groove,” or “in the zone” (Martin and Jackson, 2008). Flow is a multidimensional concept. Csikszentmihalyi’s (1990) original conceptualization of flow consists of nine dimensions. According to this framework (Nakamura and Csikszentmihalyi, 2009), three dimensions are preconditions for flow to occur: (1) “challenge-skill balance,” which refers to the perceived balance between the situational challenge represented by the activity and the personal skills necessary to overcome it; (2) “unambiguous feedback,” which concerns the clear and immediate feedback regarding the activity’s failure or success; and (3) “clear goals,” which relates to the clarity of what was expected to complete the task. The other six flow dimensions describe the flow state experience itself: (4) “action-awareness merging,” which alludes to the automaticity of actions with no separation of the self from the task; (5) “concentration on task at hand,” which refers to the absorption in the activity; (6) “sense of control,” which describes the feeling of being in control of the activity; (7) “loss of self-consciousness,” which relates to the total immersion of the person in the task to the point of forgetting oneself and becoming one with the activity; (8) “transformation of time,” which refers to the time alteration perceived during the activity; and (9) “autotelic experience,” which expresses the intrinsically rewarding feeling of the overall experience. The flow dimensions may be more or less prominent according to situational and personal factors. For instance, in some sport performances (e.g., figure skating or running) the dimensions “loss of self-consciousness” and “transformation of time” correlate weakly with other flow dimensions (Jackson, 1992; Jackson and Eklund, 2002; Stavrou et al., 2007). In music making, “loss of self-consciousness” and “transformation of time” were also found to be the weakest contributors to the flow state whereas “challenge-skill balance,” “sense of control,” and “autotelic experience” were considered to be the strongest flow state contributors (Sinnamon et al., 2012; Marin and Bhattacharya, 2013; Wrigley and Emmerson, 2013). Recently, researchers also reported that “action-awareness merging” had the lowest correlation with the other dimensions for musicians while performing in an orchestra (Cohen and Bodner, 2019a). These findings further highlight the importance of assessing the flow construct at the level of the nine dimensions, as opposed to a global flow assessment, in order to fully understand the nature of the flow experience (Jackson and Eklund, 2002).

Experiencing flow may promote musicians’ daily practice commitment (O’Neill, 1999; Araújo and Hein, 2019), may be positively related to musical creativity (MacDonald et al., 2006), and could improve music performance quality (Kirchner et al., 2008; Clark et al., 2014; Iusca, 2015). Recently, the literature discussed the idea that flow state elicitation may be used to offset the MPA negative effect (Cohen and Bodner, 2019b; Moral-Bofill et al., 2022). Small to moderate negative correlations between dispositional flow (i.e., “the frequency with which people experience flow,” Jackson et al., 1998, p. 360) and general MPA level among music students have been reported (Kirchner et al., 2008; Cohen and Bodner, 2021). Also, moderate negative correlations were found between flow state and state anxiety among music students (Fullagar et al., 2013) and between flow state and MPA symptoms among classical orchestral musicians (Spahn et al., 2021). A limitation of these studies is the use of a global score to measure the flow experience. To our knowledge, only one study (Butzer et al., 2016) investigated the correlation between the score difference (after minus before a yoga intervention) in general MPA level of music students and the score difference in the different components of dispositional flow in different musical contexts (practice, group, and solo performance). In this study, only weak to moderate significant negative correlations were obtained for the changes in the dimensions “challenge-skill balance,” “clear goals,” “action-awareness merging,” “sense of control,” “loss of self-consciousness,” and “autotelic experience” with the general MPA level during a practice performance. Weak to moderate significant negative correlations were also observed between the general MPA level and all flow dimensions except “transformation of time” during solo performance. “Action-awareness merging” and “sense of control” had the strongest significant correlations with the general MPA level. Three studies have measured musicians’ flow state during performances in front of an audience (Fullagar et al., 2013; Wrigley and Emmerson, 2013; Spahn et al., 2021). However, these studies did not compare musicians’ flow state experiences during the public performances to their flow state experiences during practice or rehearsal. To date, no study has compared flow state experiences in different performing contexts.

In sum, it is unknown how the general MPA level and the presence of an audience affect musicians’ flow state at the level of its nine dimensions. We aimed to fill this research gap. University music students ranging in their general MPA level from very low to very high performed solo without an audience (private performance session) and in front of an audience (public performance session). Based on the above mentioned social self-preservation theory and the models of social anxiety, we hypothesized that the levels of “challenge-skill balance,” “clear goals,” “action-awareness merging,” “concentration on task at hand,” “sense of control,” and “loss of self-consciousness” would be significantly lower during the public performance session than the private performance session. Furthermore, we expected this decrease to be greater in musicians with higher general MPA level than in musicians with lower general MPA level. We further predicted that the level of “autotelic experience” would be lower during the public performance session than the private performance session because an autotelic experience is an intrinsically rewarding experience and might indirectly index the absence of anxiety. We supposed this effect would be larger for music students with higher general MPA level than for music students with lower general MPA level. For the dimensions “unambiguous feedback” and “transformation of time,” we did not have strong theoretical or empirical evidence to support any predictions regarding the effects of audience and general MPA level; we thus treated these questions as exploratory issues.

## Materials and methods

These data are part of a larger research project on the psychophysiology of music performance anxiety and music performance quality [Further information on this project can be found in Guyon et al. (2020b)].

### Participants

The study used a convenience sample of 121 music students between 18 and 35 years old from Swiss music universities. All were enrolled in a classical music program following the Bologna process (3 years for Bachelor studies; 2 years for Master studies; minimum 3 years for Doctoral studies). Further sample characteristics are given in Table 1.

**Table 1:** Sample and predictors descriptive statistics.

	*M*	*SD*	Min-Max	Skewness	Kurtosis
Age	24.3	3.2	18–33	0.3	2.7
General MPA level	47.7	11.1	27–76	0.0	2.2
Depressive symptoms	10.0	7.8	0–33	1.0	3.4
Years of practice	15.4	4.1	2–22	−0.8	3.6
Hours of daily practice	4.5	1.7	1–11	0.7	4.4
Number of solo performances	8.7	11.5	0–100	4.8	34.9
Number of ensemble performances	13.3	17.3	0–100	3.3	15.1
Time difference	67.8	64.0	6–425	2.4	10.7
Preparation	1.4	1.3	0–7	1.6	6.3
	**N**			**N**	
*Gender*					
Men	52		Women	69	
*Language of the questionnaires*					
English	13		French	108	
*Instruments*					
Accordion	1		Oboe	12	
Bassoon	6		Piano	14	
Cello	11		Saxophone	2	
Clarinet	7		Trombone	4	
Doublebass	4		Trumpet	5	
Flute	7		Viola	3	
Guitar	5		Violin	13	
Horn	4		Voice	23	
*Academic year*					
Year 1	23		Year 5	22	
Year 2	18		Year 6	3	
Year 3	16		Year 7	6	
Year 4	33				
*Performance session order*					
Private – public	57		Public – private	64	

Years of practice = years spent playing their instruments. Hours of daily practice = hours spent daily to practice their instruments. Number of solo performances = number of solo performances done during the previous year. Number of ensemble performances = number of ensemble performances done during the previous year. Time difference = days between the habituation session and the first performance session. Preparation = hours spent to practice the piece between the first and the second performance.

The ethical committee of the canton of Vaud, Switzerland, approved the study (protocol number 2019–01222). All students read and signed a consent form before participating. They received a compensation of 250 Swiss francs upon study completion.

### Procedure

Music students learned about the study through social media platforms and the website of the HEMU–Haute Ecole de Musique. All participants, who expressed their willingness to participate in the study by writing to us, received a personal link to an online entry questionnaire that was used to assess sociodemographic, academic, music, and health related data as well as their general MPA level. A total of 217 music students contacted us, of whom 34 stopped replying to our e-mails after answering the online entry questionnaire. Participants were excluded based on the following criteria (number of excluded participants in parentheses): outside of the age range (2), not from a classical music program (7), playing harp, percussion or an instrument that is not part of an orchestra (5), using any drugs including recreational drugs and medication except hormonal contraception (3), suffering from diseases affecting the cardiovascular, nervous, and endocrine systems (5), having a high score for panic disorder (8) or eating disorders (7) on the Patient Health Questionnaire, being pregnant (0), lactating (0), working night shifts (0), and wearing a pacemaker (1). Three appointments were scheduled at our laboratory with the participants: a habituation session followed by two solo performance sessions. Due to the impossibility of finding appointment dates, 21 more music students (including 5 after participating at the habituation session) were excluded. The habituation session was intended to familiarize the participants with the experimental procedure and the different physiological devices by wearing them while sitting and performing. Moreover, a list of instrument specific pieces to choose from was given to the participants. The pieces are part of the standard repertoire usually required for exams, auditions, and competitions (see Guyon et al., 2020b for further information). After the habituation session, we excluded 3 music students because they reported a level of high discomfort while wearing one of the physiological devices. The two performance sessions followed the same procedure. For each performance session, participants were required to perform the same self-chosen music piece without piano accompaniment. The piece duration ranged from 2 min 36 s to 8 min 31 s (*M* = 4 min 10s, *SD* = 45 s; see Supplementary Table S1 for the complete list of the pieces). For one performance session, music students performed alone (private session), whereas for the other performance, they performed in front of an audience (public session). The audience consisted of the experimenter and five to seven music connoisseurs including two music experts who rated the quality of the participants’ performance. The performance session order (i.e., private-public and public-private) was counterbalanced across participants. The two performance sessions took place 2 days apart at the same time of the day in the afternoon. One week after the second performance session, participants received a final online questionnaire, which assessed depressive symptoms. The study was conducted in French or in English, depending on participants’ preference.

### Measurements

All questionnaires were administrated with the software EFS Survey (© UNIPARK & QuestBack, Germany).

#### General music performance anxiety level

The general MPA level was measured with the state form of the State–Trait Anxiety Inventory (for the English version Spielberger, 1989; for the French version Bruchon-Schweitzer and Paulhan, 1993). This scale contains 20 items (e.g., “I am tense”) rated on a 4-point Likert scale (1 “not at all” to 4 “very much so”). The total score ranges from 20 (no anxiety) to 80 (severe anxiety). Because we define general MPA level as the general tendency to experience anxiety during solo music performances, the instructions were adapted accordingly. Participants had to answer each item by referring to how they generally feel when they perform solo (see, e.g., Haccoun et al., 2020 for a similar approach). The Cronbach’s alpha of this scale in the present study was 0.93 for the English version and 0.92 for the French version.

#### Flow state dimensions

Immediately after each performance, participants filled in the 36-item Flow State Scale-2 (for the English version Jackson and Eklund, 2002, for the French version Fournier et al., 2007). This scale quantifies each of the following flow state dimensions (four items per dimension): challenge-skill balance (e.g., “I was challenged, but I believed my skills would allow me to meet the challenge”), unambiguous feedback (e.g., “It was really clear to me how I was going”), clear goals (e.g., “I knew clearly what I wanted to do”), action-awareness merging (e.g., “I did things correctly without thinking about trying to do so”), concentration on task at hand (e.g., “My attention was focused entirely on what I was doing”), sense of control (e.g., “I had a sense of control over what I was doing”), loss of self-consciousness (e.g., “I was not concerned with what others may have been thinking of me”), transformation of time (e.g., “Time seemed to alter (either slowed down or speeded up)”), and autotelic experience (e.g., “I really enjoyed the experience of what I was doing”). For each item, participants expressed their level of agreement on a 5-point Likert scale from 1 “strongly disagree” to 5 “strongly agree” by referring to the just-completed performance. For each dimension, a mean score ranging from 1 to 5 was computed. A higher mean score indicates a higher level of the dimension. The Cronbach’s alphas of these nine subscales are reported in the Supplementary Table S2.

#### Depressive symptoms

We assessed depressive symptoms because they have been shown to influence psychophysiological responses to stressors (Kibler and Ma, 2004; Burke et al., 2005; Hamer et al., 2007) and to correlate significantly with measures of MPA (Nielsen et al., 2018). We measured depressive symptoms with the Beck Depression Inventory-II (for the English version Beck et al., 1996; for the French version Editions du Centre de Psychologie Appliquée, 1998). This scale consists of 21 items with four sentences coded from 0 (less close to depression, e.g., “I do not feel sad”) to 3 (closest to depression, e.g., “I am so sad or unhappy that I cannot stand it”). The total score can range from 0 (no depressive symptoms) to 63 (severe depressive symptoms). The Cronbach’s alpha of this scale in the present study was 0.83 for the English version and 0.88 for the French version.

#### Sociodemographic, academic, and music data

The participants indicated their age, their gender, the name of their music university, the department, the current academic year, the main instrument, the year they started playing/studying their main instrument, the average number of public solo and ensemble performances given during the past 12 months, and the average number of hours of music practice per day.

#### Health-related data

Participants completed the Patient Health Questionnaire (for the English version Spitzer et al., 1999; for the French version Carballeira et al., 2007) to assess panic disorder and eating disorders. In addition, participants needed to list any known disease and any acute or chronic medication intake. It was required for participants to indicate if they wear a pacemaker, smoke, or take recreational drugs. Women needed to inform us whether they were pregnant or lactating.

#### Preparation time

At the end of the second performance, participants indicated how much time they had spent preparing the piece since the end of their first performance (i.e., during the last 48 h).

### Statistical analyses

Statistical analyses were performed with STATA version 16.0 for Windows (Stata Statistical Software; StataCorp LP, College Station, TX). The alpha level was considered significant below 0.05 for all tests.

#### Preliminary analyses

We tested the relationships between general MPA level and other personal variables prior to the main analyses. For continuous variables (i.e., depressive symptoms, age, number of years and hours of instrument practice, number of solo and ensemble performances), we computed pairwise correlations and for categorical variables (i.e., gender, academic level, instrument type), we performed least-squares linear regressions. We conducted these analyses to determine which variables should be included in the main analyses as control variables.

#### Main analyses

Two three-level linear mixed models with restricted marginal maximum likelihood estimation and heterogeneous residual variance structure were fitted for each of the nine flow dimensions. All models included random intercepts for participants and for session.

Model 1 tested the main effect of the two main factors, namely general MPA level and session (private vs. public). The general MPA level was treated as continuous variable. The performance session order was also included as control variable (order 1: private – public vs. order 2: public – private). As the number of days between the habituation session and the first performance session differed between participants (see Table 1), we controlled the variable “time difference” expressed in days. The variable “preparation,” defined as the amount of preparation time in hours between the first and the second performance session, was included to control for the additional preparation time for the second performance. This variable was computed as follows: First, we attributed the value 0 to the first performance session and the number of practice hours reported by the participants (e.g., 2 h) to the second performance session. Second, we centered to the individual mean (e.g., 1 h). Finally, gender and depressive symptoms were also added (see 3.2 Preliminary analyses). All continuous predictors were mean-centered.

Model 2 was an extension of model 1 including the main interaction general MPA level × session and the following control interactions: general MPA level × order, order × session, gender × order, gender × session, depressive symptoms × order, depressive symptoms × session, time difference × order, and time difference × session. Diagnostics for residuals and random effects showed that overall distributional assumptions were met for all models implying satisfactory model specification. Nevertheless, we identified a number of outliers defined as standardized residuals smaller than -3 and larger than 3. To evaluate their impact on the results, models 1 and 2 were tested again after dropping the outliers.

## Results

### Descriptive statistics

Descriptive statistics for the predictors are given in Table 1. For the outcome variables, please see Table 2.

**Table 2:** Descriptive statistics for the outcome variables.

Flow state dimensions	Private session					Public session				
	*M*	*SD*	Min-max	Skewness	Kurtosis	*M*	*SD*	Min-max	Skewness	Kurtosis
Challenge-skill balance	3.75	0.75	1.5–5	−0.6	3.3	3.73	0.75	1.5–5	−0.5	3.3
Unambiguous feedback	3.89	0.58	2.25–5	−0.5	3.1	3.78	0.67	1.75–5	−0.3	3.3
Clear goals	3.64	0.79	1.75–5	−0.4	2.6	3.62	0.80	1.5–5	−0.3	2.8
Action-awareness merging	3.51	0.67	1.75–5	−0.5	2.9	3.44	0.72	1.25–5	−0.3	2.8
Concentration on task at hand	3.33	1.08	1–5	−0.3	2.1	3.52	1.00	1–5	−0.3	2.1
Sense of control	3.31	0.85	1.25–5	−0.4	2.5	3.10	0.92	1–5	−0.0	2.5
Loss of self-consciousness	3.96	1.08	1–5	−1.0	3.1	3.12	1.12	1–5	0.1	1.9
Transformation of time	2.84	1.05	1–5	0.1	2.4	3.25	1.05	1–5	−0.5	2.6
Autotelic experience	3.48	0.94	1.5–5	−0.3	2.1	3.47	0.96	1–5	−0.3	2.5

### Preliminary analyses

The results are given in the Supplementary Tables S3–S6. There was a significant gender difference in general MPA level (*F* (1,119) = 10.09, *p* = 0.002). Female participants’ general MPA level was significantly higher than male participants’ general MPA level (mean difference = 6.23, *SE* = 1.96). Depressive symptoms and general MPA level were significantly correlated (*r* = 0.27, *p* = 0.003). There were no significant differences in general MPA level as a function of academic level and instrument. There were also no significant correlations between general MPA level and years of practice, number of solo performance, number of ensemble performance, time difference, and preparation. Based on these results, gender and depressive symptoms were entered into the main analyses to control for their potential confounding effects.

### Main analyses

The estimated models for the nine flow dimensions with all observations are reported in Tables 3–5. The models without the outliers are given in the Supplementary Tables S7–S9.

**Table 3:** Estimated linear mixed models for the dimensions “challenge-skill balance,” “unambiguous feedback,” and “clear goals.”

		Challenge-skill balance						Unambiguous feedback						Clear goals					
		*Model 1*			*Model 2*			*Model 1*			*Model 2*			*Model 1*			*Model 2*		
		Coeff.	*SE*	*p*	Coeff.	*SE*	*p*	Coeff.	*SE*	*p*	Coeff.	*SE*	*p*	Coeff.	*SE*	*p*	Coeff.	*SE*	*p*
*Main effects*																			
General MPA level		−0.01	0.01	0.16	−0.00	0.01	0.77	**−0.01**	**0.00**	**0.009**	−0.00	0.01	0.58	−0.01	0.01	0.14	−0.01	0.01	0.29
Session		−0.04	0.06	0.50	0.03	0.13	0.82	**−0.12**	**0.06**	**0.034**	−0.02	0.11	0.87	−0.04	0.07	0.62	0.26	0.15	0.083
Order		−0.04	0.12	0.74	0.11	0.19	0.58	−0.11	0.10	0.24	0.13	0.16	0.42	−0.15	0.12	0.21	0.44	0.20	0.026
Preparation		**0.10**	**0.03**	**0.002**	0.08	0.05	0.092	0.05	0.03	0.077	−0.00	0.05	0.99	**0.09**	**0.04**	**0.020**	−0.04	0.06	0.49
Gender		0.20	0.13	0.12	0.37	0.20	0.070	0.01	0.10	0.89	0.10	0.16	0.55	**0.30**	**0.13**	**0.023**	0.58	0.20	0.005
Depressive symptoms		**−0.02**	**0.01**	**0.046**	−0.01	0.01	0.25	−0.01	0.01	0.20	−0.01	0.01	0.28	−0.00	0.01	0.77	0.01	0.01	0.48
Time difference		0.01	0.01	0.39	0.01	0.01	0.43	0.01	0.01	0.46	−0.00	0.01	0.82	−0.01	0.01	0.62	−0.01	0.01	0.57
*Interactions*																			
General MPA level × order					−0.01	0.01	0.63				−0.00	0.01	0.72				−0.00	0.01	0.82
General MPA level × session					−0.00	0.01	0.62				**−0.01**	**0.01**	**0.025**				0.01	0.01	0.40
Order × session					−0.09	0.19	0.65				−0.27	0.17	0.11				**−0.64**	**0.22**	**0.004**
Gender × order					−0.24	0.27	0.37				−0.23	0.21	0.26				**−0.66**	**0.26**	**0.013**
Gender × session					−0.06	0.13	0.68				0.10	0.12	0.43				0.11	0.16	0.50
Depressive symptoms × order					−0.01	0.02	0.55				−0.00	0.01	0.99				−0.01	0.02	0.48
Depressive symptoms × session					0.01	0.01	0.48				0.00	0.01	0.52				−0.01	0.01	0.46
Time difference × order					0.01	0.02	0.78				0.01	0.02	0.65				−0.02	0.02	0.28
Time difference × session					−0.01	0.01	0.52				0.01	0.01	0.26				0.02	0.01	0.15

Model 1 tested the main effect of our factors and Model 2 tested the interactions of our factors. Significant main effects in Model 1 and significant interactions in Model 2 are written in bold. Reference categories for categorical predictors were as follows: session: private performance session; order: private performance session first – public performance session second; gender: women. For continuous predictors, coefficients express the change in the outcome measure per unit (unit for Preparation is hour and unit for Time difference is day).

**Table 4:** Estimated linear mixed models for the dimensions “action-awareness merging,” “concentration on task at hand,” and “sense of control.”

		Action-awareness merging						Concentration on task at hand						Sense of control					
		*Model 1*			*Model 2*			*Model 1*			*Model 2*			*Model 1*			*Model 2*		
		Coeff.	*SE*	*p*	Coeff.	*SE*	*p*	Coeff.	*SE*	*p*	Coeff.	*SE*	*p*	Coeff.	*SE*	*p*	Coeff.	*SE*	*p*
*Main effects*																			
General MPA level		−0.01	0.01	0.07	−0.01	0.01	0.098	**−0.02**	**0.01**	**0.008**	−0.01	0.01	0.26	**−0.02**	**0.01**	**0.001**	−0.02	0.01	0.019
Session		−0.08	0.07	0.25	−0.12	0.14	0.39	0.17	0.11	0.099	0.18	0.22	0.41	**−0.20**	**0.09**	**0.032**	−0.12	0.19	0.53
Order		0.08	0.11	0.44	0.15	0.18	0.39	−0.08	0.15	0.61	0.04	0.26	0.89	0.21	0.12	0.078	0.51	0.21	0.014
Preparation		0.01	0.04	0.79	−0.00	0.06	0.99	**0.13**	**0.06**	**0.017**	0.15	0.09	0.077	**0.16**	**0.05**	**0.001**	0.13	0.07	0.090
Gender		0.11	0.11	0.36	0.07	0.18	0.71	0.08	0.16	0.62	0.31	0.26	0.23	**0.27**	**0.13**	**0.032**	0.50	0.20	0.015
Depressive symptoms		−0.00	0.01	0.57	−0.01	0.01	0.44	**−0.02**	**0.01**	**0.042**	−0.03	0.02	0.04	−0.01	0.01	0.21	−0.01	0.01	0.65
Time difference		−0.00	0.01	0.96	0.00	0.01	0.68	**0.03**	**0.01**	**0.026**	0.03	0.02	0.10	0.01	0.01	0.19	0.00	0.01	0.75
*Interactions*																			
General MPA level × order					0.01	0.01	0.45				0.00	0.02	0.98				0.02	0.01	0.12
General MPA level × session					−0.00	0.01	0.78				−0.01	0.01	0.27				−0.01	0.01	0.15
Order × session					−0.04	0.21	0.86				0.07	0.32	0.84				−0.20	0.28	0.47
Gender × order					−0.14	0.23	0.55				−0.34	0.33	0.30				−0.47	0.25	0.059
Gender × session					0.14	0.15	0.33				−0.09	0.23	0.69				0.06	0.20	0.74
Depressive symptoms × order					0.01	0.01	0.55				−0.00	0.02	0.94				−0.01	0.02	0.68
Depressive symptoms × session					0.00	0.01	0.78				0.02	0.01	0.095				−0.01	0.01	0.60
Time difference × order					−0.03	0.02	0.071				0.01	0.03	0.68				0.02	0.02	0.26
Time difference × session					0.01	0.01	0.52				−0.01	0.02	0.53				−0.00	0.01	0.84

Model 1 tested the main effect of our factors and Model 2 tested the interactions of our factors. Significant main effects in Model 1 and significant interactions in Model 2 are written in bold. Reference categories for categorical predictors were as follows: session: private performance session; order: private performance session first – public performance session second; gender: women. For continuous predictors, coefficients express the change in the outcome measure per unit (unit for Preparation is hour and unit for Time difference is day).

**Table 5:** Estimated linear mixed models for the dimensions “loss of self-consciousness,” “transformation of time,” and “autotelic experience.”

		Loss of self-consciousness						Transformation of time						Autotelic experience					
		*Model 1*			*Model 2*			*Model 1*			*Model 2*			*Model 1*			*Model 2*		
		Coeff.	*SE*	*p*	Coeff.	*SE*	*p*	Coeff.	*SE*	*p*	Coeff.	*SE*	*p*	Coeff.	*SE*	*p*	Coeff.	*SE*	*p*
*Main effects*																			
General MPA level		**−0.02**	**0.01**	**0.032**	0.00	0.01	0.88	0.01	0.01	0.16	−0.01	0.01	0.63	**−0.01**	**0.01**	**0.034**	−0.01	0.01	0.47
Session		**−0.90**	**0.12**	**<0.001**	−0.65	0.22	0.003	**0.47**	**0.11**	**<0.001**	−0.29	0.21	0.17	−0.03	0.09	0.75	0.32	0.19	0.084
Order		**0.41**	**0.16**	**0.013**	0.96	0.28	<0.001	−0.03	0.17	0.88	−1.01	0.28	<0.001	0.00	0.14	0.99	0.36	0.24	0.14
Preparation		0.06	0.06	0.32	−0.08	0.09	0.37	**−0.17**	**0.06**	**0.003**	0.11	0.08	0.19	0.06	0.05	0.19	−0.04	0.07	0.56
Gender		0.29	0.18	0.096	0.35	0.28	0.21	0.21	0.19	0.26	−0.11	0.29	0.71	0.23	0.15	0.14	0.41	0.25	0.096
Depressive symptoms		**−0.03**	**0.01**	**0.021**	−0.02	0.02	0.27	−0.00	0.01	0.68	−0.02	0.02	0.35	−0.01	0.01	0.13	−0.01	0.01	0.58
Time difference		0.02	0.01	0.20	0.02	0.02	0.20	0.01	0.01	0.32	−0.01	0.02	0.79	0.01	0.01	0.54	0.00	0.02	0.94
*Interactions*																			
General MPA level × order					−0.01	0.02	0.72				0.03	0.02	0.14				0.00	0.01	0.89
General MPA level × session					**−0.03**	**0.01**	**0.002**				0.01	0.01	0.52				−0.01	0.01	0.17
Order × session					**−0.76**	**0.33**	**0.019**				**1.44**	**0.31**	**<0.001**				**−0.55**	**0.28**	**0.045**
Gender × order					−0.40	0.36	0.27				0.61	0.38	0.10				−0.18	0.31	0.57
Gender × session					0.36	0.23	0.13				−0.02	0.22	0.93				−0.14	0.20	0.49
Depressive symptoms × order					0.00	0.02	0.84				0.02	0.02	0.44				−0.02	0.02	0.31
Depressive symptoms × session					−0.02	0.01	0.18				−0.00	0.01	0.91				0.01	0.01	0.68
Time difference × order					0.03	0.03	0.24				0.02	0.03	0.53				0.01	0.03	0.62
Time difference × session					**−0.04**	**0.02**	**0.036**				0.02	0.02	0.15				0.01	0.01	0.70

Model 1 tested the main effect of our factors and Model 2 tested the interactions of our factors. Significant main effects in Model 1 and significant interactions in Model 2 are written in bold. Reference categories for categorical predictors were as follows: session: private performance session; order: private performance session first – public performance session second; gender: women. For continuous predictors, coefficients express the change in the outcome measure per unit (unit for Preparation is hour and unit for Time difference is day).

#### Effects of general MPA level, session, and their interaction

**Figure 1:**
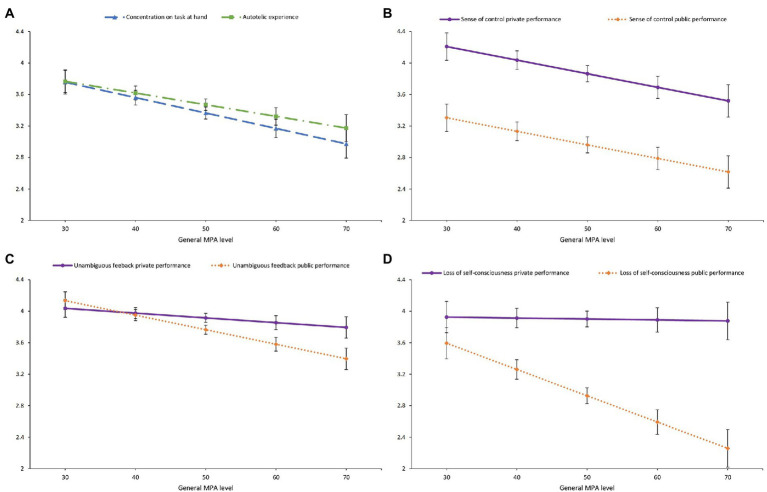
Model-predicted marginal means along the general MPA level for **(A)** “Concentration on task at hand” and “Autotelic experience,” **(B)** “Sense of control” for the private and for the public performance sessions, **(C)** “Unambiguous feedback” for the private and for the public performance sessions, **(D)** “Loss of self-consciousness” for the private and for the public performance sessions.

In model 1, a significant general MPA level effect was present for “unambiguous feedback,” “concentration on task at hand,” “sense of control,” “loss of self-consciousness,” and “autotelic experience” (see Figure 1). With increasing general MPA level, the level of these five dimensions decreased significantly. There was a significant main effect of session for “unambiguous feedback,” “sense of control,” and “loss of self-consciousness” (see Figure 1). The level of these three dimensions was significantly higher during the private performance session than the public performance session.

In model 2, a significant interaction between the general MPA level and session was observed for “unambiguous feedback” and “loss of self-consciousness” (see Figure 1). Post-hoc contrast analyses showed that the level of “unambiguous feedback” was higher for the private performance session than for the public performance session for general MPA levels higher than 47 (*p*s = 0.002–0.049). The effect of the general MPA level was not significant for the private performance session (coefficient = −0.01, *SE* = 0.01, *p* = 0.31) and significant for the public performance session (coefficient = −0.02, *SE* = 0.01, *p* = 0.001). The level of “loss of self-consciousness” was significantly higher for the private performance session than for the public performance session for general MPA levels higher than 32 (*p*s = <0.001–0.050). The effect of the general MPA level was not significant for the private performance session (coefficient = −0.00, *SE* = 0.01, *p* = 0.89) and significant for the public performance session (coefficient = −0.03, *SE* = 0.01, *p* < 0.001).

#### Effects of the control variables

Participants with order 1 (private–public) had a significantly higher level of “loss of self-consciousness” than participants with order 2 (public–private). The levels of “challenge-skill balance,” “clear goals,” “concentration on task at hand,” and “sense of control” increased significantly with an increase in the amount of preparation, whereas the level of “transformation of time” decreased significantly with an increase in the amount of preparation. Women reported significantly lower levels of “clear goals” and “sense of control” than men. The lower the level of depressive symptoms was, the higher the levels of “challenge-skill balance,” “concentration on task at hand,” and “loss of self-consciousness.” The higher the amount of time between the habituation session and the first performance session was, the higher the level of “concentration on task at hand.”

**Figure 2:**
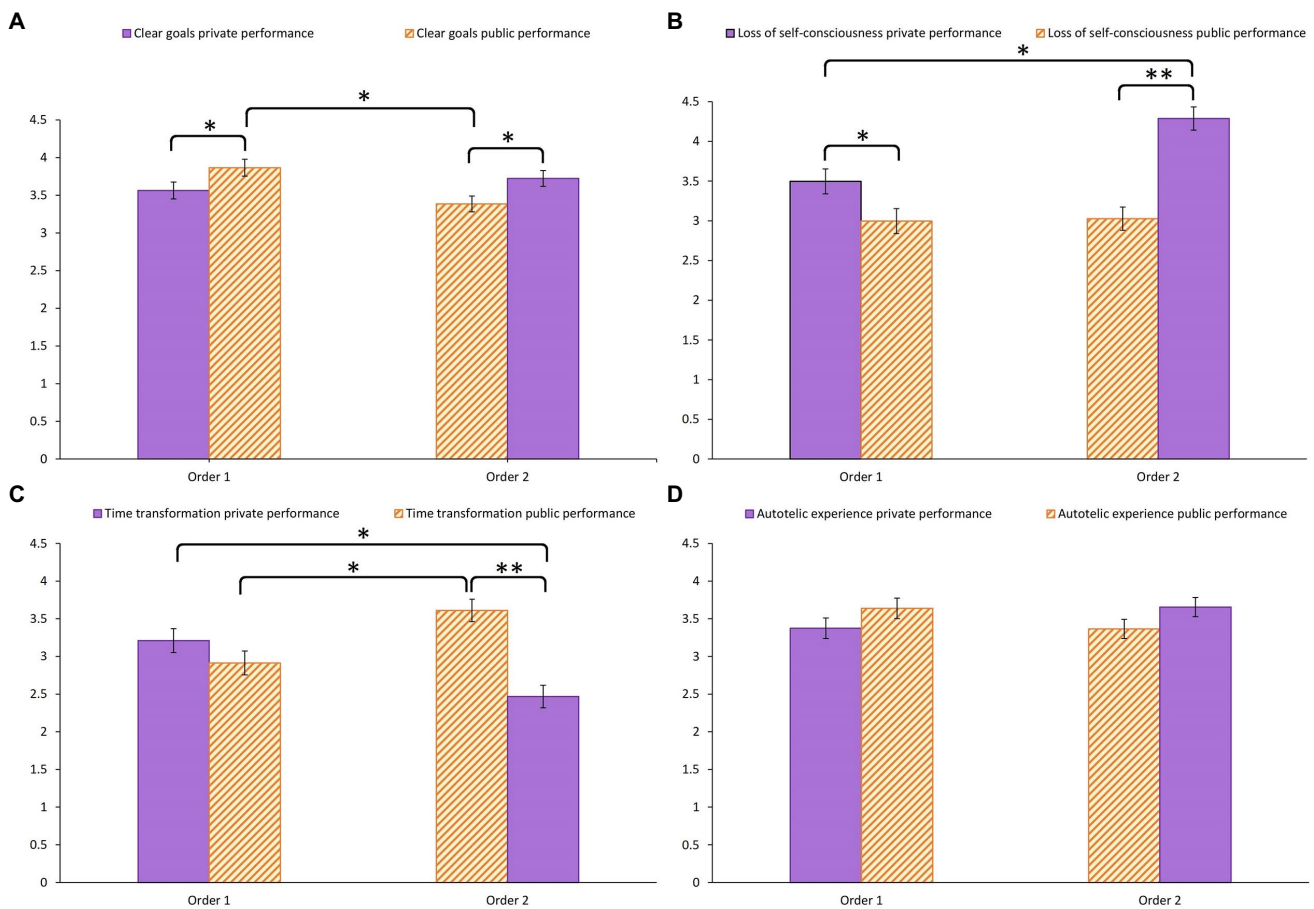
Model-predicted marginal means for order 1 (private session performed first then public session) and order 2 (public session performed first then private session) **(A)** “Clear goals,” **(B)** “Loss of self-consciousness,” **(C)** “Transformation of time,” and **(D)** “Autotelic experience.” The symbol “*” indicates a *value of p* below 0.05 and the symbol “**” indicates a *value of p* below 0.001.

A significant interaction order × session was found for “clear goals,” “loss of self-consciousness,” “transformation of time,” and “autotelic experience” (see Figure 2). To understand these interactions, we tested the session effect for each order separately and the order effect for each session separately. Moreover, we tested whether the levels of these four dimensions during the first performance and the second performance differed significantly from each other. The level of “clear goals” was higher for the public performance session than the private performance session in order 1 (coefficient = 0.30, *SE* = 0.14, *p* = 0.028), whereas the level of “clear goals” was lower for the public performance than the private performance in order 2 (coefficient = −0.34, *SE* = 0.13, *p* = 0.007). The level of “clear goals” during the public performance was lower in order 2 than in order 1 (coefficient = −0.48, *SE* = 0.16, *p* = 0.003), but the order effect was not significant for the private performance (coefficient = 0.16, *SE* = 0.16, *p* = 0.33). The level of “clear goals” was significantly lower during the first performance session than the second performance session (coefficient = −0.32, *SE* = 0.11, *p* = 0.004). The level of “loss of self-consciousness” was lower for the public performance session than the private performance session in order 1 (coefficient = −0.50, *SE* = 0.20, *p* = 0.014) and in order 2 (coefficient = −1.26, *SE* = 0.19, *p* < 0.001). The level of “loss of self-consciousness” was higher during the private performance in order 2 than during the private performance in order 1 (coefficient = 0.79, *SE* = 0.23, *p* = 0.001), but the order effect was not significant for the public performance (coefficient = 0.03, *SE* = 0.23, *p* = 0.90). The level of “loss of self-consciousness” was significantly lower during the first performance session than during the second performance session (coefficient = −0.38, *SE* = 0.16, *p* = 0.019). The level of “transformation of time” was higher for the public performance session than for the private performance session in order 2 (coefficient = 1.14, *SE* = 0.18, *p* < 0.001). No significant difference was found between the two performance sessions in order 1 (coefficient = −0.30, *SE* = 0.19, *p* = 0.12). The level of “transformation of time” during the private performance session was lower in order 2 than in order 1 (coefficient = −0.74, *SE* = 0.23, *p* = 0.001), whereas the level of “transformation of time” during the public performance session was higher in order 2 than in order 1 (coefficient = 0.70, *SE* = 0.23, *p* = 0.003). The level of “transformation of time” was significantly higher during the first performance session than the second performance session (coefficient = 0.72, *SE* = 0.15, *p* < 0.001). For both orders, the levels of “autotelic experience” for the private and public performance sessions did not significantly differ from each other (coefficient = 0.26, *SE* = 0.17, *p* = 0.13 for order 1 and coefficient = −0.29, *SE* = 0.15, *p* = 0.068 for order 2). For both performance sessions, the order effect was not significant (coefficient = 0.28, *SE* = 0.20, *p* = 0.16 for the private performance session; coefficient = −0.27, *SE* = 0.20, *p* = 0.17 for the public performance session). The level of “autotelic experience” was significantly higher during the second performance session than the first performance session (coefficient = 0.28, *SE* = 0.14, *p* = 0.045).

For “clear goals,” the interaction gender × order was significant. Men had a higher level of “clear goals” than women in order 1 (coefficient = 0.63, *SE* = 0.19, *p* = 0.001), whereas the gender difference was not significant in order 2 (coefficient = −0.03, *SE* = 0.18, *p* = 0.89). Men in order 2 had a significantly lower level of “clear goals” than men in order 1 (coefficient = −0.54, *SE* = 0.20, *p* = 0.006). In contrast, women in order 1 and women in order 2 did not significantly differ in their levels of “clear goals” (coefficient = 0.12, *SE* = 0.17, *p* = 0.46).

The interaction time difference × session was significant for “loss of self-consciousness”. The effect of time difference was significant for the private performance session (coefficient = 0.03, *SE* = 0.02, *p* = 0.034) and non significant for the public performance session (coefficient = −0.00, *SE* = 0.01, *p* = 0.97). The level of “loss of self-consciousness” during the public performance session was significantly higher than during the private performance session for all time differences (*p*s < 0.001).

#### Main analyses without outliers

Of the 968 total observations for each of the nine flow dimensions, we dropped the following number of observations considered as outliers: “challenge-skill balance,” 17 for models 1 and 2; “unambiguous feedback,” 4 for model 1, 3 for model 2; “clear goals,” 2 for model 1, 3 for model 2; “action-awareness merging,” 5 for models 1 and 2; “concentration on task at hand,” 9 for models 1 and 2; “sense of control,” 0 for models 1 and 2; “loss of self-consciousness,” 18 for models 1 and 2; “transformation of time,” 14 for model 1, 15 for model 2; and “autotelic experience,” 2 for models 1 and 2. Cronbach’s alphas were significantly increased for all dimensions after discarding the outliers (see Supplementary Table S2).

As can be seen in the Supplementary Tables S7–S9, overall, dropping the outliers did not significantly change the results compared to the analyses with all observations. Only the significant main effect of depressive symptoms for “challenge-skill balance” became non significant.

## Discussion

In this study, we investigated how music students’ flow state experience defined in terms of its nine dimensions is influenced by the audience’s presence and by students’ general MPA level. Based on the social self-preservation theory (Dickerson and Kemeny, 2004) and on empirical research on music performance (e.g., Guyon et al., 2020a), we had hypothesized that the levels of “challenge-skill balance,” “clear goals,” “action-awareness merging,” “concentration, on task at hand,” “sense of control,” “loss of self-consciousness,” and “autotelic experience” would be lower during the public performance session than during the private performance session. These hypotheses were confirmed for “sense of control” and “loss of self-consciousness.” “Sense of control” was significantly lower during the public performance than the private performance for all music students. For “loss of self-consciousness,” the session effect was significant for all music students except for those with a general MPA level lower than 32. Contrary to our hypotheses, the audience’s presence did not negatively affect music students’ perceived competence to meet the situational demands, their sense of what they want to do, their ability to act spontaneously, their focus on the task, and their enjoyment. Furthermore, an unanticipated significant session effect was found for “unambiguous feedback.” Similarly to “loss of self-consciousness,” this dimension’s level was significantly lower during the public performance than during the private performance for participants with a general MPA level higher than 47. We speculate that this effect might be partly linked to the higher self-consciousness due to the audience’s presence observed among music students with higher general MPA level compared to music students with lower general MPA level. Music students with higher general MPA level may have reduced ressources to properly monitor and evaluate their public performance’s progress due to their increased preoccupation with the audience’s judgement and the associated higher attentional focus on the audience. Finally, the level of “transformation of time” was higher during the public performance session than during the private performance session, yet, only for music students whose first performance session was public and not for music students whose first performance session was private.

Drawing from the social anxiety models (Hiemisch et al., 2002; Hofmann, 2007; Nieuwenhuys and Oudejans, 2012) and empirical research on MPA (e.g., Guyon et al., 2020a), we had also hypothesized that the above mentioned session effects would be larger for music students with higher general MPA level than for music students with lower general MPA level. We found that the general MPA level significantly affected the levels of “unambiguous feedback,” “concentration on task at hand,” “sense of control,” “loss of self-consciousness,” and “autotelic experience.” For “concentration on task at hand,” “sense of control,” and “autotelic experience,” the general MPA level had a main effect; their levels decreased significantly with increasing general MPA level independently of the performance session. In contrast, the levels of “unambiguous feedback” and “loss of self-consciousness” decreased significantly with increasing general MPA level during the public performance only. The general MPA level did not significantly modulate music students’ perceived competence to meet the situational demands, their sense of what they want to do, and their ability to act automatically. Thus, the general MPA level effect during the public performance session was found for “concentration on task at hand,” “sense of control,” “loss of self-consciousness,” and “autotelic experience” as expected and additionally for “unambiguous feedback.” Considering that “sense of control” and “autotelic experience” emerged as two of the strongest contributors to the global flow experience in music making (Sinnamon et al., 2012; Marin and Bhattacharya, 2013; Wrigley and Emmerson, 2013), the observed general MPA level effect on these two dimensions can be considered to be in agreement with the negative correlation between flow and general MPA level found in previous studies (Kirchner et al., 2008; Fullagar et al., 2013; Cohen and Bodner, 2019a). It is important to note that compared to music students with lower general MPA level, music students with higher general MPA level reported poorer focus and lower feeling of control on the task as well as less enjoyment during both performance sessions. These findings extent a previous study’s results, which showed that musicians with higher general MPA level exhibited more anxiety, more negative self-statements, and less self-efficacy than musicians with lower general MPA level not only when performing in front of an audience but also when performing alone (Craske and Craig, 1984).

The levels of “clear goals,” “loss of self-consciousness,” and “autotelic experience” were significantly higher during the second performance session than during the first performance session, whereas the level of “transformation of time” was significantly lower. These effects cannot be explained by the amount of preparation time between first and second performance sessions, as this variable was included in the models. We interpret these findings as being in line with the idea that the first performance session allowed the music students to habituate themselves to the performance situation (e.g., same experimental environment, instructions, music piece during both performance sessions; Groves and Thompson, 1970).

With increasing amount of preparation time between the two performance sessions, the levels of “challenge-skill balance,” “clear goals,” “concentration on task at hand,” and “sense of control” increased, wherease the level of “transformation of time” decreased. These findings extend previous research by Marin and Bhattacharya (2013) who showed that pianists’ daily amount of practice was positively associated with their global dispositional flow. Furthermore, Butkovic et al. (2015) have observed that the amount of weekly music practice does not correlate positively with the general flow proneness but with flow proneness that is specific to activities in the musical field.

Compared to male music students, female music students reported less “sense of control” and less “clear goals.” The majority of studies on dispositional flow among musicians did not find any significant gender effect (Marin and Bhattacharya, 2013; Habe et al., 2019; Cohen and Bodner, 2021 but see Valenzuela et al., 2018). Similarly, two studies assessing the global flow state reported no significant differences between male and female musicians (Wrigley and Emmerson, 2013; Spahn et al., 2021). Our significant gender effect for “sense of control” could be understood from the perspective of the personal control theory according to which women tend to consider that their actions have less impact on the environment than men (Ross and Mirowsky, 2002). This factor is thought to contribute to a stronger sense of uncontrollability among women (Barlow, 2002). With regard to the gender effect on “clear goals,” we caution against overinterpreting this finding because the gender difference emerged only among music students who started with the private performance session.

Finally, the more depressive symptoms music students reported, the lower were the levels of “challenge-skill balance,” “concentration on task at hand,” and “loss of self-consciousness,” independently of the performance session. These findings extend the results of Mosing et al. (2018), who found a significant, although small, negative correlation between depressive symptoms and flow proneness during music activities in the general population. Our findings are consistent with the fact that decreased ability to concentrate and low self-esteem are two of the depressive symptoms assessed with the Beck Depression Inventory-II (Beck et al., 1996; American Psychiatric Association, 2013).

The direction of the session and preparation effects on “transformation of time” was opposite to the direction of the effects observed for all other flow dimensions. Previous studies showed that “transformation of time” correlates poorly with the other flow dimensions and is the weakest predictor of the overall flow experience in music making (Sinnamon et al., 2012; Marin and Bhattacharya, 2013; Wrigley and Emmerson, 2013). We also found very low correlations between “transformation of time” and all other dimensions during both performance sessions (see Supplementary Table S3). Tempo and rhythm and thus accurate time awareness play a prominent role in music making. A good music performance hinges on respecting the tempo and rhythm requirements of the music piece. Thus, having the sense that time passes in a way that is different from normal during a music performance does not seem to be a desirable experience for a musician. Knowing that the flow state positively correlates with optimal performance (Jackson and Csikszentmihalyi, 1999; Norsworthy et al., 2017), it seems reasonable to conclude that during music performances low levels of “transformation of time” would be more consistent with the flow experience than high levels.

### Limitations

The inclusion of a habituation session in the study procedure is a strength of the present study. Yet, due to several factors (i.e., Covid-19 pandemic, personal issues, rescheduling of the appointments), the time between the habituation session and the first performance session was longer than originally planned (around 30 days) for numerous participants (mean of 68 days). In addition, participants were allowed to play/sing freely (e.g., any pieces with or without scores) during the habituation session. This was not the case during the performance sessions. These factors may have contributed to a weaker habituation effect than initially anticipated and indirectly, at least partly, to the above mentioned significant differences between first and second performance sessions obtained for four flow dimensions. Future studies should organize a habituation session closer in time to the performance sessions and reproduce more closely the conditions of the performance sessions.

As it can be seen in the Supplementary Table S1, Cronbach’s alphas were good to excellent for seven of the nine flow dimensions. Yet, for “unambiguous feedback” and “action-awareness merging” they were lower than 0.70 (for all subjects and the French version). The validation of the French version of the FSS-2 by Fournier et al. (2007) was conducted with a sample of athletes. The flow experience of athletes and musicians may be different (Habe et al., 2019). It might therefore be important to conduct a validation study with French-speaking musicians as well to determine if the reliability of the subscales assessing these two dimensions can be improved.

Finally, this study only looked at music students’ flow experience in two contexts of short music performances in the laboratory. To generalize these results, it would be necessary to investigate if the effects found in this study are observed in other more naturalistic contexts (e.g., actual audition or concert) during longer performances as well as in different musician populations (e.g., musicians who finished their studies or jazz musicians).

## Conclusion and implications

In conclusion, we obtained the following main findings. Music students reported less sense of control during the public performance session than the private performance session. Performance feedback was more ambiguous during the public performance than the private performance for participants with a general MPA level higher than 47. The level of “loss of self-consciousness” was lower during the public performance than the private performance for participants with a general MPA level higher than 32. The levels of “concentration on task at hand,” “sense of control,” and “autotelic experience” decreased significantly with increasing general MPA level during both performance sessions. The levels of “unambiguous feedback” and “loss of self-consciousness” decreased significantly with increasing general MPA level during the public performance only.

The findings of the present study enhance theory by acquiring a more comprehensive understanding of the processes that underpin individual reactions to social-evaluative stressors in performance situations. Furthermore, the present results have the potential to guide the development and implementation of theory-led interventions. First, taken together the results show that the effects of audience and general MPA level vary greatly across the nine flow dimensions. This supports the recommendation, in line with the original multidimensional perspective on flow, that whenever possible the flow experience should be analyzed at the level of its nine dimensions rather than as a global score (Jackson and Eklund, 2002). Second, the study significantly contributes to the literature on the negative psychological effects of social-evaluative stressors (Dickerson and Kemeny, 2004) by showing which flow dimensions are affected by the audience’s presence. The latter had a negative effect on “sense of control” for all music students and on “loss of self-consciousness” for almost all music students. Thus, increasing sense of control and reducing self-consciousness during public performances by means of appropriate interventions would be a goal to pursue for any musician. Third, the study contributes to expanding our understanding of MPA and its relationship to flow. Considering individual differences in terms of general MPA level is crucial to better understand musicians’ flow experience at the level of its nine dimensions level. Based on the present findings, musicians with a general MPA level higher than 47 should benefit from interventions aiming to improve their ability to effortlessly process clear and unambiguous performance-related information, to be totally focused on and connected to the musical task without having extraneous thoughts, and to enjoy themselves while making music. In a recent study, music professors and students followed a psychological program combining emotional awareness and regulation exercises, mindfulness exercises, practice and performance preparation, and quick self-regulation exercises (Moral-Bofill et al., 2022). After completing this program, music professors and students reported significantly lower general MPA levels as well as significantly higher levels of “action-awareness merging,” “sense of control,” and “loss of self-consciousness” during a public solo performance. The dimensions “concentration on task at hand,” “transformation of time,” and “autotelic experience” did not show a significant change. This result seems promising, but the dimensions “challenge-skill balance,” “unambiguous feedback,” and “clear goals” were not assessed. Furthermore, a study shows that after completing an intervention containing traditional yoga postures, breathing techniques, and meditation, the musicians showed no significant improvement in any dispositional flow dimension but only a significant increase in the global flow score (Butzer et al., 2016). Future research should investigate how different interventions influence both state and dispositional measures of flow dimensions. It is further suggested that addressing depressive symptoms should contribute to the improvement of musicians’ flow experience. Before recommending any specific interventions, these should be properly evaluated.

Building on the findings of the present study, future research could investigate the (causal) relationship between the flow state experience at the level of its nine dimensions and meaningful well-being, health, and performance outcomes.

## Data availability statement

The raw data supporting the conclusions of this manuscript will be made available by the authors, without undue reservation, to any qualified researcher.

## Ethics statement

The studies involving human participants were reviewed and approved by the ethical committee of the canton of Vaud, Switzerland. The patients/participants provided their written informed consent to participate in this study.

## Author contributions

All authors listed have made a substantial, direct, and intellectual contribution to the work and approved it for publication.

## Funding

The Swiss National Science Foundation funded this study with a grant to Patrick Gomez (subsidy number 100019_182251).

## Conflict of interest

The authors declare that the research was conducted in the absence of any commercial or financial relationships that could be construed as a potential conflict of interest.

## Publisher’s note

All claims expressed in this article are solely those of the authors and do not necessarily represent those of their affiliated organizations, or those of the publisher, the editors and the reviewers. Any product that may be evaluated in this article, or claim that may be made by its manufacturer, is not guaranteed or endorsed by the publisher.
