# Supplementary material for: How audience and general music performance anxiety affect classical music students’ flow experience: A close look at its dimensions
Source: Front Psychol. 2022 Oct 28;13:959190. doi: 10.3389/fpsyg.2022.959190 (PMC9649719; doi:10.3389/fpsyg.2022.959190)
Supplement: Supplementary file 1 [file Data_Sheet_1.PDF]

Supplementary Materials

1 Supplementary Data S1. List of pieces performed by the participants ordered by instrument with the number of participants performing each piece

Number of participants	Music pieces performed
	**Accordion**
1	Haydn – Sonata in D major Hob XVI 37 – 1st movement Allegro con brio – without repetitions
	**Bassoon**
6	Mozart – Concerto for bassoon in B-flat major K.191 – 1st movement Allegro – Bars 35-152
	**Cello**
6	Haydn – Cello concerto n°1 in C major Hob.VIIb.1 – 1st movement Allegro – Bars 22-89
5	Haydn – Cello concerto n°2 in D major Hob.VIIb.2 – 1st movement Allegro moderato – Bars 29-106
	**Clarinet**
7	Mozart – Clarinet concerto in A major K.622 – 1st movement Allegro – Bars 57 - 192
	**Double Bass**
3	Dittersdorf – Concerto for double bass n°2 Kr.172 – 1st movement Allegro moderato with cadenza by Gruber
1	Vanhal – Concerto for double bass in D major – 1st movement Allegro moderato
	**Flute**
2	Mozart – Flute concerto n°1 in G major K.313 – 1st movement Allegro maestoso – Bars 31-149
5	Mozart – Flute concerto n°2 in D major K.314 – 1st movement Allegro aperto – Bars 32-151
	**Guitar**
1	Craeyvanger – Introduction and variations on a theme from the Opera "Der Freischütz" from Von Weber
1	Giuliani – Concerto for guitar n°1 in A major, Op. 30 – 1st movement Allegro maestoso – Bars 106-206
1	Matiegka – Guitar sonata, Op.23
1	Coste – La source du Lyson, Op.47 – Rondeau villageois Allegretto
1	Sor – Fantaisie N°6 ('Les Adieux') for Guitar, Op. 21. – Bars 1-68
	**Horn**
3	Mozart – Horn concerto n°2 in E flat major KV.417 – 1st movement Allegro maestoso
1	Mozart – Horn concerto n°4 in E flat K.495 – 1st movement Allegro moderato – Bars 36-189
	**Oboe**
12	Mozart – Oboe Concerto in C major K.314/271k – 1st movement Allegro aperto

	**Piano**
1	Haydn – Sonata n°60 in C major Hob.XVI.50 – 1st movement Allegro – Bars 1-53 & 102-150 - without repetitions
1	Mozart - Rondo in D major k485
1	Mozart – Sonata n°6 in D major K.284/205b – 1st movement Allegro –without repetitions
2	Mozart – Sonata n°7 in C major K.309/284b – 1st movement Allegro con spirito – without repetitions
1	Mozart – Sonata n°8 in A minor K.310/300d – 1st movement Allegro maestoso – without repetitions
4	Mozart – Sonata n°10 in C major K.330/300h – 1st movement Allegro moderato – without repetitions
1	Mozart – Sonata n°12 in F major K.332 – 1st movement Allegro – Bars 1-176 – without repetitions
3	Mozart – Sonata n°14 in C minor K.457 – 1st movement Molto allegro – without repetitions
	**Saxophone**
1	Telemann – Fantasia n°2 in A minor Grave - Vivace - Adagio – Allegro
1	Telemann – Fantasia n°10 in F-sharp minor A Tempo giusto – Presto – Moderato
	**Trombone**
2	David – Concertino for trombone in E-flat major – 1st movement Allegro maestoso
2	Handel – Concerto in F minor – 2nd and 4th movements Allegro
	**Trumpet**
3	Haydn – Concerto for trumpet in E-flat major Hob.VIIe:1 – 1st movement Allegro
2	Hummel – Trumpet concerto in E-flat major S.49 – 1st movement Allegro con spirito – Bars 66-224
	**Viola**
3	Hoffmeister – Viola concerto in D major – 1st movement Allegro
	**Violin**
1	Bach – Partita no°2 in D minor – Allemande
2	Mozart – Violin concerto n°3 in G major K.216 – 1st movement Allegro – Bars 38-192
4	Mozart – Violin concerto n°4 in D major K.218 – 1st movement Allegro – Bars 42-177
6	Mozart – Violin concerto n°5 in A major K.219 – 1st movement Allegro aperto – Bars 40-139
	**Voice**
	Baritone
2	Mozart – Die Zauberflöte – Ein Mädchen oder Weibchen
	Countertenor
1	Mozart – La Clemenza di Tito – Deh per questo istante –Adagio only
	Mezzo-Soprano
1	Mozart – Ascanio in Alba - Ah di sì nobil alma
	Soprano
3	Mozart – Così fan tutte - Una donna a quindici anni

1	Mozart - Così fan tutte – Ah, scostati! ... Smanie implacabili
2	Mozart – Dans un bois solitaire
3	Mozart – Die Zauberflöte - Ach ich fühl’s, es ist verschwunden!
1	Mozart – Don Giovanni - Batti, batti oh bel Masetto
1	Mozart - Le nozze di Figaro - Dove sono i bei momenti - without recitativo
5	Mozart - Le nozze di Figaro - Giunse alfin il momento...Deh vieni, non tardar
2	Mozart - Le nozze di Figaro - Venite inginocchiatevi
	Tenor
1	Mozart - Le nozze di Figaro – Un’ aura amorosa

3

4

Supplementary Table S2. Cronbach's alphas for all nine flow dimensions.

	Private performance session						Public performance session					
	All subjects		English version		French version		All subjects		English version		French version	
	All observations	w.o. outliers	All observations	w.o. outliers	All observations	w.o. outliers	All observations	w.o. outliers	All observations	w.o. outliers	All observations	w.o. outliers
**CS**	0.81	0.89	0.91		0.80	0.89	0.84	0.89	0.87		0.84	0.89
**UF**	0.66	0.69	0.70	0.86	0.67		0.72	0.74	0.88		0.68	0.70
**CG**	0.83	0.86	0.93		0.83	0.85	0.84		0.93		0.82	
**AM**	0.68	0.72	0.85		0.63	0.68	0.69	0.72	0.86		0.66	0.70
**CT**	0.94	0.95	0.90	0.94	0.95	0.95	0.91	0.93	0.90	0.93	0.92	0.93
**SC**	0.87		0.92		0.86		0.90		0.95		0.89	
**LS**	0.87	0.89	0.84	0.87	0.88	0.89	0.88	0.90	0.83	0.83	0.87	0.90
**TT**	0.86	0.91	0.81		0.88	0.92	0.88	0.91	0.66		0.89	0.92
**AE**	0.90	0.90	0.93		0.89	0.90	0.90	0.90	0.94		0.90	0.90

Note of table 1 – w.o. outliers: Cronbach's alpha calculated excluding outliers; CS: Challenge-skill balance; UF: Unambiguous feedback; CG: Clear goals; AM: action-awareness merging; CT: Concentration on task at hand; SC: Sense of control; LS: Loss of self-consciousness; TT: Transformation of time; AE: Autotelic experience.

Supplementary Table S3. Preliminary analyses – Pearson correlations between the general MPA level and seven predictors (N = 121)

	General MPA level	Age	Depressive symptoms	Years of practice	Hours of daily practice	Number of solo performance	Number of ensemble performance	Time difference
Age	0.02							
Depressive symptoms	**0.27**	0.05						
Years of practice	-0.01	**0.44**	0.003					
Hours of daily practice	-0.04	**-0.19**	0.03	0.12				
Number of solo performance	-0.07	0.17	-0.10	-0.05	0.05			
Number of ensemble performance	-0.06	0.13	0.04	0.14	-0.002	-0.03		
Time difference	-0.11	0.12	0.08	0.09	-0.14	0.17	0.11	
Preparation	-0.03	-0.08	0.05	0.05	**0.24**	-0.08	0.13	0.11

Note of supplementary table 3 - Years of practice = years spent playing their instrument. Hours of daily practice = hours spent daily to practice their instrument. Number of solo performances = number of solo performances done during the previous year. Number of ensemble performances = number of ensemble performances done during the previous year. Time difference = number of days between the habituation session and the first performance session. Preparation = hours spent to practice the piece between the first and the second performance. Significant correlations (p < 0.05) are written in bold.

Supplementary Table S4. Preliminary analyses – Pearson correlations between the flow dimensions and individual factors included in the model for the private performance session (*N* = 121)

	CS	UF	CG	MA	CT	SC	LS	TT	AE	General MPA level	Depressive symptoms
UF	**0.35**										
CG	**0.36**	**0.55**									
MA	**0.35**	**0.38**	**0.24**								
CT	**0.49**	**0.48**	**0.42**	**0.23**							
SC	**0.45**	**0.44**	**0.46**	**0.45**	**0.60**						
LS	**0.27**	**0.20**	0.028	**0.25**	**0.27**	**0.40**					
TT	-0.08	-0.074	-0.052	0.029	-0.077	-0.17	**-0.31**				
AE	0.49	**0.43**	**0.51**	**0.48**	**0.53**	**0.66**	**0.30**	0.001			
General MPA level	**-0.23**	-0.18	-0.17	-0.16	**-0.25**	**-0.24**	-0.076	0.071	**-0.20**		
Depressive symptoms	**-0.24**	-0.15	-0.063	-0.10	**-0.28**	**-0.12**	-0.076	0.018	**-0.19**	**0.27**	
Time difference	0.085	-0.006	-0.11	-0.04	0.16	0.066	0.17	0.018	-0.022	-0.11	0.076

Note of supplementary table 4 - CS: Challenge-skill balance; UF: Unambiguous feedback; CG: Clear goals; AM: Action-awareness merging; CT: Concentration on task at hand; SC: Sense of control; LS: Loss of self-consciousness; TT: Transformation of time; AE: Autotelic experience. Time difference = number of days between the habituation session and the first performance session. Significant correlations ($p < 0.05$) are written in bold.

Supplementary Table S5. Preliminary analyses – Pearson correlations between the flow dimensions and individual factors included in the model for the public performance session (*N* = 121)

	CS	UF	CG	MA	CT	SC	LS	TT	AE	General MPA level	Depressive symptoms
UF	**0.62**										
CG	**0.59**	**0.56**									
MA	**0.39**	**0.26**	**0.25**								
CT	**0.50**	**0.57**	**0.38**	**0.26**							
SC	**0.63**	**0.59**	**0.52**	**0.47**	**0.64**						
LS	**0.39**	**0.42**	**0.24**	**0.39**	**0.41**	**0.48**					
TT	**-0.25**	**-0.29**	**-0.22**	0.051	**-0.27**	**-0.31**	-0.17				
AE	**0.60**	**0.47**	**0.51**	**0.40**	**0.46**	**0.60**	**0.36**	-0.12			
General MPA level	**-0.22**	**-0.35**	-0.16	**-0.21**	**-0.28**	**-0.40**	**-0.41**	0.13	**-0.30**		
Depressive symptoms	**-0.19**	-0.13	-0.10	-0.094	-0.13	**-0.22**	**-0.33**	0.006	-0.16	**0.27**	
Time difference	0.054	0.14	0.024	0.021	0.15	0.076	-0.015	0.068	0.068	-0.11	0.076

Note of supplementary table 5 - CS: Challenge-skill balance; UF: Unambiguous feedback; CG: Clear goals; AM: Action-awareness merging; CT: Concentration on task at hand; SC: Sense of control; LS: Loss of self-consciousness; TT: Transformation of time; AE: Autotelic experience. Time difference = number of days between the habituation session and the first performance session. Significant correlations (p < 0.05) are written in bold.

Supplementary Table S6. Preliminary analyses – One-way analyses of variances with general MPA level as dependent variable (*N* = 121)

	Sum of squares	df	Mean Square	F	*p*	R-squared
Gender	**1151.92**	**1**	**1151.92**	**10.09**	**0.002**	**0.08**
Academic level	379.74	6	63.29	0.50	0.81	0.03
Instrument	271.11	4	67.78	0.54	0.70	0.02

Note of supplementary table 6 – Academic level = current academic year of the music students. Instrument = type of the main music instrument played by the music students. The musical instruments were divided into five groups: 1) piano and accordion players, 2) woodwind players, 3) brass players, 4) string players, and 5) singers.

Supplementary Table S7. Estimated linear mixed models for the dimensions "challenge-skill balance", "unambiguous feedback", and "clear goals" excluding outliers.

	**Challenge-skill balance**						**Unambiguous feedback**						**Clear goals**					
	Model 1			*Model 2*			*Model 1*			*Model 2*			*Model 1*			*Model 2*		
	Coeff.	*SE*	*p*	Coeff.	SE	*p*	Coeff.	*SE*	*p*	Coeff.	*SE*	*p*	Coeff.	*SE*	*p*	Coeff.	*SE*	*p*
**Main effects**																		
General MPA level	-0.01	0.01	0.15	-0.00	0.01	0.74	**-0.01**	**0.00**	**0.007**	-0.01	0.01	0.47	-0.01	0.01	0.15	-0.01	0.01	0.28
Session	-0.03	0.06	0.59	0.01	0.13	0.93	**-0.12**	**0.06**	**0.039**	-0.04	0.11	0.73	-0.04	0.07	0.55	0.23	0.15	0.12
Order	-0.07	0.13	0.58	0.05	0.20	0.81	-0.11	0.10	0.26	0.10	0.16	0.53	-0.17	0.12	0.18	0.41	0.20	0.040
Preparation	**0.09**	**0.03**	**0.007**	0.10	0.05	0.059	0.05	0.03	0.088	0.01	0.04	0.82	**0.09**	**0.04**	**0.021**	-0.04	0.06	0.54
Gender	0.21	0.13	0.12	0.43	0.21	0.039	0.02	0.10	0.85	0.12	0.16	0.45	**0.30**	**0.13**	**0.024**	0.57	0.21	0.006
Depressive symptoms	-0.02	0.01	0.061	-0.01	0.01	0.26	-0.01	0.01	0.18	-0.01	0.01	0.23	-0.00	0.01	0.71	0.01	0.01	0.57
Time difference	0.01	0.01	0.47	0.01	0.01	0.53	0.01	0.01	0.55	-0.00	0.01	0.73	-0.00	0.01	0.62	-0.01	0.01	0.56
**Interactions**																		
General MPA level x order				-0.01	0.01	0.60				-0.00	0.01	0.82				-0.00	0.01	0.85
General MPA level x session				-0.00	0.01	0.63				**-0.01**	**0.01**	**0.020**				0.01	0.01	0.39
Order x session				0.01	0.19	0.94				-0.21	0.17	0.21				**-0.60**	**0.22**	**0.005**
Gender x order				-0.28	0.27	0.30				-0.25	0.21	0.25				**-0.65**	**0.26**	**0.015**
Gender x session				-0.12	0.13	0.37				0.07	0.12	0.55				0.11	0.16	0.49
Depressive symptoms x order				-0.01	0.02	0.69				0.00	0.01	1.00				-0.01	0.02	0.51
Depressive symptoms x session				0.00	0.01	0.63				0.01	0.01	0.48				-0.01	0.01	0.53
Time difference x order				0.01	0.02	0.74				0.01	0.02	0.64				-0.02	0.02	0.30
Time difference x session				-0.01	0.01	0.51				0.01	0.01	0.23				0.02	0.01	0.15

Note of supplementary table S7 – Model 1 tested the main effect of our factors and Model 2 tested the interactions of our factors. Significant main effects in Model 1 and significant interactions in Model 2 are written in bold. Reference categories for categorical predictors were as follows: session: private performance session; order: private performance session first – public performance session second; gender: women. For continuous predictors, coefficients express the change in the outcome measure per unit (unit for Preparation is hour and unit for Time difference is day).

Supplementary Table S8. Estimated linear mixed models for the dimensions "action-awareness merging", "concentration on task at hand", and "sense of control" excluding outliers

	**Action-awareness merging**						**Concentration on task at hand**						**Sense of control**					
	Model 1			*Model 2*			*Model 1*			*Model 2*			*Model 1*			*Model 2*		
	Coeff.	*SE*	*p*	Coeff.	*SE*	*p*	Coeff.	*SE*	*p*	Coeff.	*SE*	*p*	Coeff.	*SE*	*p*	Coeff.	*SE*	*p*
**Main effects**																		
General MPA level	-0.01	0.01	0.095	-0.01	0.01	0.14	**-0.02**	**0.01**	**0.008**	-0.02	0.01	0.21	**-0.02**	**0.01**	**0.001**	-0.02	0.01	0.019
Session	-0.06	0.07	0.37	-0.10	0.14	0.50	0.18	0.11	0.097	0.17	0.22	0.44	**-0.20**	**0.09**	**0.032**	-0.12	0.19	0.53
Order	0.09	0.11	0.42	0.17	0.18	0.34	-0.09	0.15	0.55	-0.00	0.27	0.99	0.21	0.12	0.078	0.51	0.21	0.014
Preparation	-0.00	0.04	1.00	-0.01	0.06	0.79	**0.14**	**0.06**	**0.017**	0.16	0.09	0.077	**0.16**	**0.05**	**0.001**	0.13	0.07	0.090
Gender	0.09	0.12	0.44	0.05	0.19	0.80	0.10	0.16	0.55	0.29	0.27	0.28	**0.27**	**0.13**	**0.032**	0.50	0.20	0.015
Depressive symptoms	-0.00	0.01	0.51	-0.01	0.01	0.43	**-0.02**	**0.01**	**0.043**	-0.03	0.02	0.048	-0.01	0.01	0.21	-0.01	0.01	0.65
Time difference	-0.00	0.01	0.91	0.01	0.01	0.66	**0.03**	**0.01**	**0.025**	0.03	0.02	0.105	0.01	0.01	0.19	0.00	0.01	0.75
**Interactions**																		
General MPA level x order				0.01	0.01	0.41				0.00	0.02	0.88				0.02	0.01	0.12
General MPA level x session				-0.00	0.01	0.53				-0.01	0.01	0.32				-0.01	0.01	0.15
Order x session				-0.06	0.21	0.77				0.07	0.33	0.84				-0.20	0.28	0.47
Gender x order				-0.14	0.24	0.55				-0.28	0.33	0.41				-0.47	0.25	0.059
Gender x session				0.15	0.15	0.30				-0.07	0.23	0.78				0.06	0.20	0.74
Depressive symptoms x order				0.01	0.02	0.63				-0.00	0.02	0.87				-0.01	0.02	0.68
Depressive symptoms x session				0.00	0.01	0.78				0.02	0.01	0.097				-0.01	0.01	0.60
Time difference x order				-0.04	0.02	0.073				0.01	0.03	0.65				0.02	0.02	0.26
Time difference x session				0.01	0.01	0.63				-0.01	0.02	0.55				-0.00	0.01	0.84

Note of supplementary table S8 - Model 1 tested the main effect of our factors and Model 2 tested the interactions of our factors. Significant main effects in Model 1 and significant interactions in Model 2 are written in bold. Reference categories for categorical predictors were as follows: session: private performance session; order: private performance session first – public performance session second; gender: women. For continuous predictors, coefficients express the change in the outcome measure per unit (unit for Preparation is hour and unit for Time difference is day).

Supplementary Table S9. Estimated linear mixed models for the dimensions "loss of self-consciousness", "transformation of time", and "autotelic experience" excluding outliers.

	**Loss of self-consciousness**						**Transformation of time**						**Autotelic experience**					
	Model 1			*Model 2*			*Model 1*			*Model 2*			*Model 1*			*Model 2*		
	Coeff.	*SE*	*p*	Coeff.	*SE*	*p*	Coeff.	*SE*	*p*	Coeff.	*SE*	*p*	Coeff.	*SE*	*p*	Coeff.	*SE*	*p*
**Main effects**																		
General MPA level	**-0.02**	**0.01**	**0.030**	0.00	0.01	0.78	0.01	0.01	0.15	-0.01	0.01	0.70	**-0.02**	**0.01**	**0.026**	-0.01	0.01	0.39
Session	**-0.94**	**0.12**	**<0.001**	-0.68	0.23	0.003	**0.46**	**0.11**	**<0.001**	-0.32	0.21	0.13	-0.03	0.09	0.77	0.34	0.19	0.073
Order	**0.47**	**0.17**	**0.006**	0.98	0.29	0.001	-0.05	0.17	0.76	1.07	0.29	0.000	0.01	0.14	0.93	0.37	0.24	0.12
Preparation	0.06	0.07	0.33	-0.06	0.09	0.53	**-0.18**	**0.06**	**0.002**	0.11	0.08	0.17	0.07	0.05	0.18	-0.05	0.07	0.52
Gender	0.29	0.18	0.11	0.42	0.29	0.15	0.21	0.19	0.27	-0.12	0.30	0.69	0.22	0.15	0.15	0.38	0.25	0.12
Depressive symptoms	**-0.03**	**0.01**	**0.024**	-0.02	0.02	0.25	-0.01	0.01	0.60	-0.02	0.02	0.27	-0.01	0.01	0.13	-0.01	0.01	0.60
Time difference	0.02	0.01	0.21	0.02	0.02	0.21	0.02	0.01	0.27	-0.00	0.02	0.90	0.01	0.01	0.54	0.00	0.02	0.94
**Interactions**																		
General MPA level x order				-0.01	0.02	0.64				0.03	0.02	0.15				0.00	0.01	0.86
General MPA level x session				**-0.04**	**0.01**	**0.002**				0.01	0.01	0.61				-0.01	0.01	0.23
Order x session				**-0.68**	**0.34**	**0.049**				**1.50**	**0.31**	**<0.001**				**-0.59**	**0.28**	**0.036**
Gender x order				-0.40	0.37	0.28				0.64	0.38	0.092				-0.16	0.31	0.62
Gender x session				0.23	0.24	0.35				-0.04	0.22	0.86				-0.12	0.20	0.53
Depressive symptoms x order				0.01	0.02	0.80				0.02	0.02	0.41				-0.02	0.02	0.29
Depressive symptoms x session				-0.02	0.02	0.24				0.00	0.01	0.99				0.00	0.01	0.71
Time difference x order				0.04	0.03	0.22				0.02	0.03	0.50				0.01	0.03	0.64
Time difference x session				**-0.04**	**0.02**	**0.037**				0.02	0.02	0.18				0.01	0.01	0.70

Note of supplementary table S9 - Model 1 tested the main effect of our factors and Model 2 tested the interactions of our factors. Significant main effects in Model 1 and significant interactions in Model 2 are written in bold. Reference categories for categorical predictors were as follows: session: private performance session; order: private performance session first – public performance session second; gender: women. For continuous predictors, coefficients express the change in the outcome measure per unit (unit for Preparation is hour and unit for Time difference is day).
